# Modeling long‐distance dispersal of emerald ash borer in European Russia and prognosis of spread of this pest to neighboring countries within next 5 years

**DOI:** 10.1002/ece3.4437

**Published:** 2018-08-24

**Authors:** Marina J. Orlova‐Bienkowskaja, Andrzej O. Bieńkowski

**Affiliations:** ^1^ A.N. Severtsov Institute of Ecology and Evolution Russian Academy of Sciences Moscow Russia

**Keywords:** *Agrilus planipennis*, ash pest, biological invasions, emerald ash borer, simulation model

## Abstract

**Aim:**

To develop an approach to model the spatial dynamics of emerald ash borer *Agrilus planipennis* (Coleoptera: Buprestidae) in European Russia. This tree‐killing pest was detected in Moscow 15 years ago and began to spread, posing a threat to ashes all over Europe. The aim was to determine its probable current range and to evaluate the probability of its dispersal to neighboring countries within the next 5 years.

**Location:**

Cities and transport hubs of European Russia and neighboring countries. Ash trees in this region occur mainly in urban plantations and along highways.

**Methods:**

Pairwise distances between all locations were used as the main parameter determining the probability of pest spread. For each location, the probability of detection of *A. planipennis* was calculated using three simulation recurrent models of long‐distance dispersal. Parametrization was made by comparison with results of surveys in 2003–2015. Field data on the range of *A. planipennis* in 2016‐2017 were mapped and used for model verification. A prognosis of spread of the pest by 2022 was made.

**Results:**

A model based on fat‐tailed kernel corresponds to both negative and positive results of surveys. According to the model, the current range is likely to be restricted to Russia, but probability of detection of the pest in the east of Belarus, Ukraine, Estonia, Latvia, and Lithuania by 2022 is 15%–40%.

**Main conclusions:**

The forestry services of neighboring countries probably have about 5 years to prepare for the invasion of this pest, but regular surveys are necessary, since the pest can appear at any time. The case considered shows that the simple approach based on a fat‐tailed kernel and just one parameter—pairwise distance between cities—can be used for modeling long‐distance dispersal of alien pests of urban plantations.

## 1 INTRODUCTION

The spread of alien invasive tree pests is a serious conservation problem, as these species pose a threat to the biodiversity of forests and artificial plantations (Freer‐Smith & Webber, 2017). Timely, accurate, and focused action can potentially reduce the negative impact of the pests, so understanding the population dynamics and spread of invasive pests has become one of the major challenges of the 21st century (Leung, Cacho, & Spring, 2010). It is impossible to survey in all locations; however, reliable data on the range can be obtained by developing probabilistic models of distribution based on limited number of field observations (Yemshanov, Koch, Barry Lyons, Ducey, & Koehler, 2012). These models allow an evaluation of the current ranges of pests and can be used to predict future dynamics of these ranges.

Delimitating of ranges of invasive species is a difficult task. First, the species spread in heterogeneous landscape, and the heterogeneity is often unknown. Second, it is difficult to localize the epicenter of invasion in many cases. Third, the mechanisms of spread are often unknown. There are several approaches that solve this problem in cases of severe uncertainty (Leung et al., 2010; Yemshanov et al., 2012). But in the case of spatial dynamics of the emerald ash borer in European Russia, the uncertainty is lower, because some a priori information is known: (a) This species is connected almost exclusively with urban plantations (Straw, Williams, Kulinich, & Gninenko, 2013), (b) the epicenter of invasion is Moscow (Baranchikov, Mozolevskaya, Yurchenko, & Kenis, 2008), and (c) the main mechanism of long‐distance dispersal is an unintentional introduction by people (Volkovitsh & Mozolevskaya, 2014). We have developed a simple approach to model the spatial dynamics of spread of this alien pest in European Russia.

**Figure 1:**
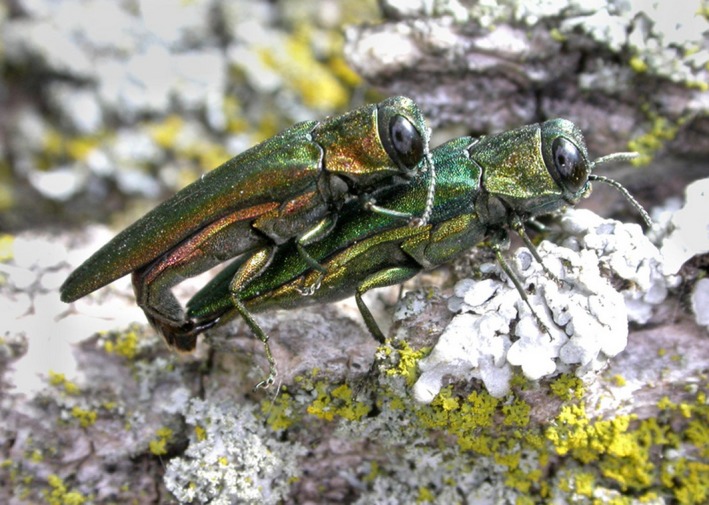
Emerald ash borer *Agrilus planipennis* adults (photograph by David Cappaert, http://www.forestryimages.org)

Emerald ash borer *Agrilus planipennis* (Coleoptera: Buprestidae) (Figure 1) is native to East Asia and is one of the most destructive forest pest in North America (Herms & McCullough, 2014). In 2003, it was first detected in Europe in Moscow city and then caused great damage to ash trees in 11 regions of Russia (Baranchikov et al., 2008; Straw et al., 2013; Orlova‐Bienkowskaja, 2014). This pest poses a serious threat to urban ash plantations all over Europe. Destruction of these plantations can potentially reduce the quality of life in the cities. So the expansion of EAB range attracts attention not only of entomologists, ecologists, and experts in forestry, but also of ordinary people. The data on its current range are scarce, and no reliable prognosis of further spread of the pest has been made. So an evaluation of the current range of the pest and a prognosis of its spread is of great importance.

The only model of *A. planipennis* range in Europe was made using MaxEnt analysis (Flø, Krokene, & Økland, 2015). This analysis attempted to show the potential geographic distribution of *A. planipennis* in Europe based primarily on climatic variables. This model has three significant flaws. First, the data of surveys by Orlova‐Bienkowskaja (2014a,b) used as a base for this model were misunderstood: All surveyed locations were regarded as a locations of detection of *A. planipennis*, although in fact the survey in many locations gave negative results. Second, the model is based on the assumption that *A. planipennis* is spreading in the forests, although in fact it almost never occurs in the forests in European Russia (Baranchikov et al., 2008; Volkovitsh & Mozolevskaya, 2014). Third, location data from the native range of the pest have not been used to parametrize the model. The output of the model suggests that the only European areas that are at threat from *A. planipennis* are the areas surrounding the currently known locations of the pest.

A number of models have been developed to predict the spread of *A. planipennis* in North America (Iverson, Prasad, Bossenbroek, Sydnor, & Schwartz, 2010; Kovacs et al., 2010; Muirhead et al., 2006; Yemshanov et al., 2015). These models (especially the work by Yemshanov et al. (2015)) have been the inspiration for the current work. But these models cannot be directly applied to European Russia, as they describe dispersal of the pest in the continuous forest area, not from city to city. Ecology of *A. planipennis* in these regions is quite different. *Agrilus planipennis* is a forest pest in North America, but in European Russia, this pest of ash occurs only in urban areas or near highways or railways. The main host plant in European Russia is *Fraxinus pennsylvanica* which were introduced from North America and planted in cities and along the roads.

The current situation with EAB in Europe is quite different from the situation in North America. While *A. planipennis* is a forest pest in the USA and Canada (Herms & McCullough, 2014), it is almost exclusively a pest of artificial plantations in Russia (Selikhovkin, Popovichev, Mandelshtam, Vasaitis, & Musolin, 2017). The infestations of the only native ash species *F. excelsior* have been recorded only near severely damaged plantations of *F. pennsylvanica* (Baranchikov, Seraya, & Grinash, 2014; Smirnov, 2014). *Agrilus planipennis* has not become a forest pest even in Voronezh region (Blummer & Shtapova, 2016), where *Fraxinus excelsior* is widespread in the forests and *A. planipennis* has appeared for at least 12 years (Baranchikov, Demidko, & Seraya, 2016).

Research to date suggests natural spread of *A. planipennis* populations is limited to only a few kilometers per year (Siegert, McCullough, Williams, Fraser, & Poland, 2010). In contrast, long‐distance spread resulting from anthropogenic movement of infested ash material, such as nursery trees, can greatly increase local and regional rate of spread through the formation of satellite populations (Siegert, McCullough, Liebhold, & Telewski, 2014). Current data of dendrochronological analysis support the suggestion that the only entry point of invasion to the continent was Moscow and the pest spread to other regions from Moscow (Baranchikov et al., 2016). In just 10 years after the first record in Europe, *A. planipennis* has been detected as far as 460 km from the initial infestation (Orlova‐Bienkowskaja, 2014b). This fast spread can be explained only by long‐distance dispersal, that is, dispersal by unintentional introduction by humans. Unlike the United States, ash firewood is not used in European Russia. So it is suspected that the vector of dispersal is hitchhiking of adult beetles on vehicles (Straw et al., 2013). Our study as some other current research of North American range (Yemshanov et al., 2015) focuses exclusively on long‐distance dispersal of *A. planipennis* and does not address aspects of biological (i.e., local) spread. We have developed the model of the current range of *A. planipennis* and made a prognosis of its spread by 2022.

## 2 METHODS

**Figure 2:**
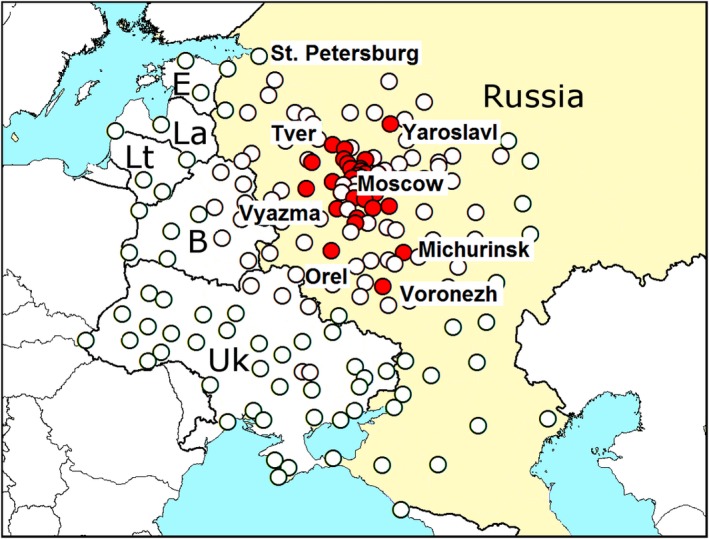
Cities and transport hubs of Russia, Belarus, Ukraine, Estonia, Latvia, and Lithuania used as a base for the model for modeling of range of *Agrilus planipennis*. *Red dots*—cities, where *A*. *planipennis* has been detected. *White dots*—other cities . B, Belarus; E, Estonia; La, Latvia; Lt, Lithuania; Uk, Ukraine

We have chosen 173 cities and transport hubs of Russia, Belarus, Ukraine, Estonia, Latvia, and Lithuania as a base for the model (Figure 2). The list of these locations with their coordinates is given in Supporting information Appendix S1 (list “Pairwise distances”). The large cities and transport hubs are concentrated near Moscow. *Agrilus planipennis* is difficult to detect: it often remains unnoticed for several years after the infestation (Siegert, Mercader, & McCullough, 2015). So our models do not calculate the probability of introduction or establishment of the pest in each city, but the probability of detection of *A*. *planipennis* if a survey is conducted. This approach allows us to compare calculated probabilities with the data of surveys.

Our model, like some others (e.g., Yemshanov et al., 2015), does not take into account the climatic factors. The main parameter determining the probability of spread of *A. planipennis* from infested city *I* to another city *J* is the distance between them (d_*ij*_). The pairwise distances between all locations were calculated using the formula for calculating of distances between geographical locations (Mikhailov, Kudryavtsev, & Davydov, 2009):

(1) $$\begin{array}{cc} d_{\mathrm{ij}} & =\text{arccos}(\text{sin}\left({\text{lat}}_{i}\right)*\text{sin}\left({\text{lat}}_{j}\right)+\\ & \text{cos}\left({\text{lat}}_{i}\right)*\text{cos}\left({\text{lat}}_{j}\right)*\text{cos}\left({\text{lon}}_{i}-{\text{lon}}_{j}\right)) \end{array}$$

where lat_*i*_ and lon_*i*_ are latitude and longitude of location *I*, and lat_*j*_ and lon_*j*_ are latitude and longitude of location *J*. The table of all pairwise distances has been compiled (Supporting information Appendix S1: list “Pairwise distances”).

Let P_*ij*_ be of the annual probability of the dispersal of *A. planipennis* from the infested city *I* to the destination *J* which is situated at the distance d_*ij*_ from it. Then,

(2) $$P_{\mathrm{ij}}=f\left(d_{\mathrm{ij}}\right)$$

P_*ij*_ ranges from 0 to 1. We presume that the function *f*(*d*) is the same for all locations in the territory under examination. The same assumption was made in some models of spread of *A. planipennis* in North America (Kovacs et al., 2010). Because we assume that the geographical extent of our study is very large, and that the spatial resolution exceeds the species’ dispersal distance by biological means, we have further made the simplifying assumption that the *P*_*ij*_ values are independent of the likelihood of arrival at adjacent locations within 1 year (which we believe is a fair assumption when considering long‐distance, human‐mediated spread). The same assumption was made by Yemshanov et al. (2015) for modeling of dispersal of *A. planipennis* in North America.

The likelihood that *A. planipennis* will be *not* introduced from the city *I* to city *J* this year is 1‐P_*ij*_. The likelihood that *A. planipennis* will be *not* introduced to the city *J* from any other city is production of likelihoods of these independent events: $\underset{i}{\Pi }\left(1-P_{\mathrm{ij}}\right)$. Therefore, the annual probability of spread of *A. planipennis* to the city *J* can be calculated as follows:

(3) $$P_{j}=1-\underset{i}{\Pi }\left(1-P_{\mathrm{ij}}\right)$$

*Agrilus planipennis* was first recorded in Europe in 2003. The first infested city was Moscow (Volkovitsh & Mozolevskaya, 2014). It is the starting point of our model. At first, we calculate the likelihood of detection of *A. planipennis* for each city in 2004 using the table of pairwise distances and the annual likelihood of detection of *A. planipennis* calculated with the Equations (2) and (3). Then, we calculate the likelihood of detection of *A. planipennis* for each city in 2005 taking into account the calculated probability of its spread in 2004:

(4) $$P_{ji2005}=P_{i2004}*f\left(d_{\mathrm{ij}}\right)$$

Combining the Equations (3) and (4), we calculate the likelihood of its infestation from any location in 2005:

(5) $$P_{j2005}=1-\underset{i}{\Pi }\left(1-P_{i2004}*f\left(d_{\mathrm{ij}}\right)\right)$$

Then, in the same way, we calculated *P*_*j*2006_ using *P*_*j*2005_ and so on. This recurrent algorithm allowed us to calculate likelihood of infestation in any year up to 2017 (See Supporting information Appendices S1, S2 and S3). The function *f*(*d*) is unknown. But it is obvious that (a) the closer the two cities are the higher is the probability of spread of the pest from one city to another; (b) although the greater the distance between two cities the lower the probability of spread of pest from one city to another, this probability never reaches zero. This kind of distribution is described as a negative exponential kernel (Equation (6)), normal kernel (Equation (7)), or Cauchy (fat‐tailed) kernel (Equation (8)).

(6) $$P_{d}=e^{-\alpha d}$$

(7) $$P_{d}=e^{-\beta d^{2}}$$

(8) $$P_{d}=\;\frac{1}{1+{\left(\frac{d}{\gamma }\right)}^{2}}$$

A negative exponential kernel was used for modeling of spread of *A. planipennis* in North America by Muirhead et al. (2006) and Kovacs et al. (2010). A normal kernel is the most often used function for description of stochastic processes. A fat‐tailed distribution is often used for modeling of long‐distance dispersal (Leung et al., 2010). So we have elaborated three models based on these three kernels correspondingly: E‐model, N‐model, and C‐model.

Parameters α, β, and γ are the constants which are determined as a result of parametrization of the models. The models were parametrized by the least squares method. As the likelihood of infestation is calculated for 2015, we parametrize the model, that is, chose the parameter of the kernel so that the calculated probabilities of infestation correspond to the observed infestations in the best way. For this purpose, we assign “1” to locations where *A. planipennis* has been found and “0” to the locations where a survey was performed but *A. planipennis* was not found. We have 26 locations where *A. planipennis* was found by 2015 and 26 locations where surveys did not reveal signs of *A. planipennis* (Supporting information Appendices S1, S2 and S3, lists “2015”). The parameters α, β, and γ were selected so that the sum of squares of all differences between the calculated likelihoods and assigned values was minimal:

*α* = 0.0459
*β* = 0.000747
*γ* = 10.125

**Figure 3:**
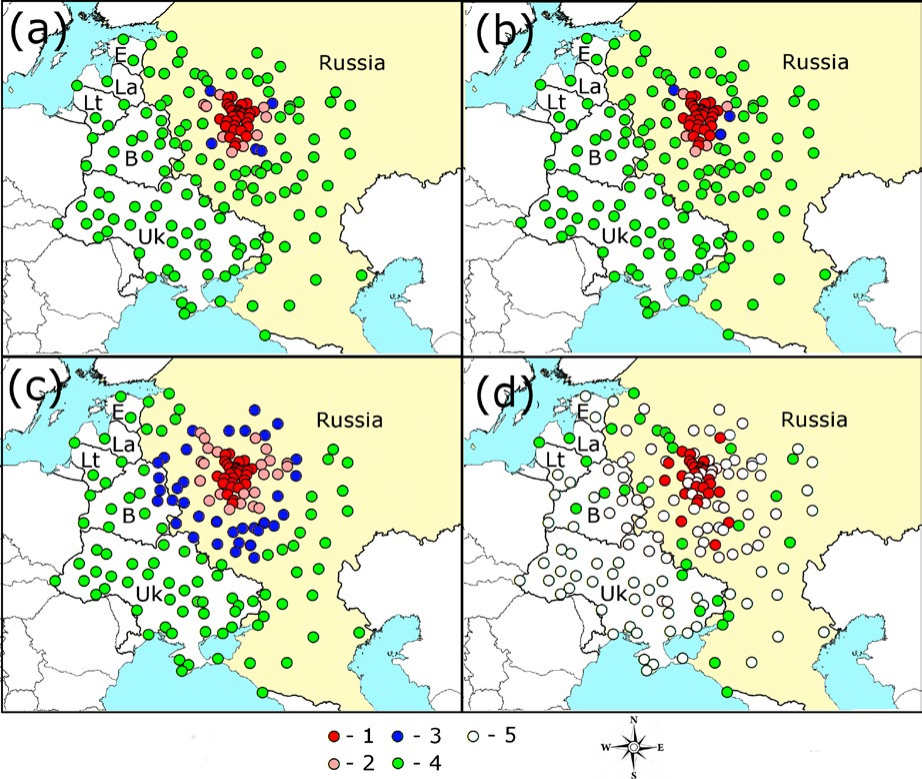
a, b, c—probabilities of detection of *Agrilus planipennis* by 2017 by E‐model, N‐model, and C‐model correspondingly, d—data of field surveys. 1—large cities and transport hubs, where probability of detection of *A. planipennis* by 2017 is more than 85% (maps a–c) or it has been detected (in map d), 2—probability is 40%–85%, 3—probability is 15%–40%, 4—less than 15% (maps a–c) or *A. planipennis* has not been detected (last surveys in 2016–2017), 5—no data of surveys. B, Belarus; E, Estonia; La, Latvia; Lt, Lithuania; Uk, Ukraine

The probability of detection of *A. planipennis* by 2017, calculated by each model, was verified by comparison with field data of surveys. Field data on the range of *A. planipennis* known by 2017 are mapped (Figure 3d). The following sources of information about surveys were used: Baranchikov (2013), Baranchikov and Kurteev (2012), Baranchikov, Gninenko, and Yurchenko (2010), Martynov and Nikulina (2016), Martynov, Nikulina, and Foroschuk (2016), Martynov, Nikulina, and Shokhin (2017), Orlova‐Bienkowskaja (2014a,b), Peregudova (2016), Rosselkhoznadzor (2014), Shankhiza (2007), Selikhovkin et al. (2017), Straw et al. (2013), Volkovitsh and Mozolevskaya (2014), own unpublished data and personal communications by Y.N. Kovalenko, A.V. Prisnyj, S.K. Ryndevich, M.E. Smirnov, A.I. Miroshnikov, D.M. Musolin, A.B. Ruchin, I.A. Zabaluev, R.N. Ishin, A.N. Drogvalenko, and A. Bukejs.

The model which corresponds to the data of surveys better than others was chosen. A prognosis of the spread of *A. planipennis* by 2022 was made on the basis of this model. The maps were created in ArcGIS 10.4.1 in Albers equal‐area conic projection, because this projection gives an idea of the area occupied by the pest.

## 3 RESULTS

### 3.1 Current range and verification of the models

For each locality, the probability of detection of *A. planipennis* was calculated by E‐model, N‐model, and C‐model and compared with field survey data (Figure 3, Table 1). Results of field surveys of *A. planipennis* (Supporting information Appendix S4) are used for verification of the models. It is very unlikely that *A*. *planipennis* would disappear in any locality, where it was detected, but it could appear in any locality, where it was previously absent. So we used all positive results (localities, where *A. planipennis* was detected in 2003–2017) and current negative results (localities, where *A. planipennis* was not detected during surveys in 2016–2017).

**Table 1:**
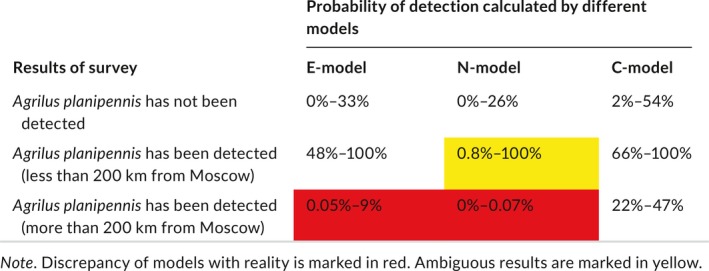
Verification of the models by results of surveys

Cities and transport hubs concentrated near Moscow became infested by *A. planipennis* quickly. Spread beyond the Moscow region has been slower. This change in pest spread is supported by field data and simulated by all models. All models fit well to the negative results of the surveys. In all locations where surveys had not revealed signs of *A. planipennis* by 2017, the calculated probability was lower than 54%. Detection of *A. planipennis* in all surveyed localities situated less than 200 km from Moscow also corresponds to all models. But E‐model and N‐model fail to simulate the observed detections of *A. planipennis* at a distance more than 200 km from Moscow. In contrast, C‐model fits well to the observed detection of *A. planipennis* far from Moscow. The calculated likelihoods of detection in these remote locations calculated by this model are 22%–47%.

There are more pink and blue circles in the map in Figure 3c than in the maps in Figures 3a and 2b. This means that the likelihood of detection of the pest in many localities calculated by C‐model is between 15% and 85%. In other words, this model has a higher level of uncertainty. This makes it more realistic, as the level of uncertainty in the prognosis of spread of invasive organisms is also very high (Yemshanov et al., 2012). However, it does not mean that such a prognosis is useless. If the likelihood of infestation of the location is between 15% and 85%, it means that an urgent survey is necessary to reveal the real situation.

Thus, C‐model fits well to data of field survey. According to this model, current range of *A. planipennis* is probably restricted to European Russia. The maximal calculated probability of detection of this pest outside the country is in the east of Belarus: Mogilev Region (probability is up to 21%), Vitebsk Region (up to 17%), and in the north of the Ukraine: Sumy Region (up to 16%) and Kharkiv Region (up to 11%). Probability of detection of *A. planipennis* in each city is indicated in Supporting information Appendix S3 (list “2017”).

### 3.2 Prognosis of spread by 2022

**Figure 4:**
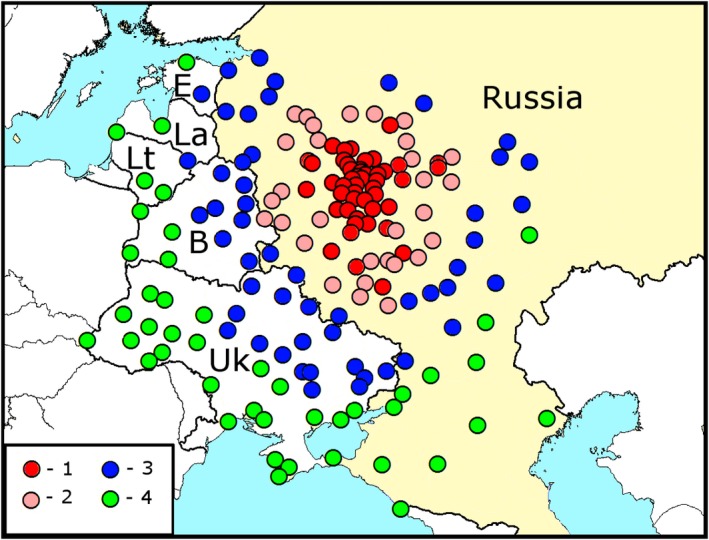
Prognosis of spread of *Agrilus planipennis* by 2022 by C‐model. 1—cities and transport hubs, where probability of detection is >85%. 2—probability is 40%–85%. 3—probability is 15–40%. 4—probability is <15%. B, Belarus; E, Estonia; La, Latvia; Lt, Lithuania; Uk, Ukraine

As C‐model seems to be more relevant for modeling long‐distance dispersal of *A. planipennis*, it was used for forecasting pest spread. For calculating the probability of pest detection in 2018–2022, we used the same recurrent algorithm as for previous years (Supporting information Appendix S5). To take into account the data of field surveys 2003–2017, we assigned “1” instead of calculated probabilities of detection to all localities, where *A. planipennis* was detected. The results of forecasting are presented in the map (Figure 4).

The model demonstrates that whether the rate of spread of *A. planipennis* remains unchanged of the next few years, by 2022 the pest could be detected all over European Russia. The probability of its detection in the cities and transport hubs of eastern parts of Belarus, Ukraine, Estonia, Latvia, and Lithuania is up to 15%–40%. The probability of detection of the pest in the western parts of these countries or in the Caucasus is less than 15%.

## 4 DISCUSSION

It is unsurprising that C‐model corresponds to the data of surveys better than two others. It is known that the relevance of models of range expansion is extremely sensitive to the precise shape of the redistribution kernel and, in particular, to the tail of the distribution and that fat‐tailed kernels describe the process of range expansion better than others (Kot, Lewis, & van den Driessche, 1996).

An outbreak of *A. planipennis* in the countries neighboring with European Russia in the near future seems to be inevitable. The forestry services of Belarus, Ukraine, Estonia, Latvia, and Lithuania have probably about 5 years to prepare for invasion of this pest. This conclusion may seem obvious without modeling, but it is not true. First, the speed of spread of species cannot be correctly estimated on the intuitive level or using the model of constant‐speed traveling waves (Kot et al., 1996). Second, the speed of spread of different invasive pests is quite different. For example, leaf beetle *Luperomorpha xanthodera* (Fairmaire, 1888) was first recorded in Europe in 2003 (i.e., at the same time with EAB). But it is spreading much faster and has now occupied almost the whole Europe—from Spain to Russia (Bieńkowski & Orlova‐Bienkowskaja, 2018). Our study has shown that EAB is spreading slower.

By bad luck, there are no significant geographic barriers which could slow the spread of the pest to the neighboring countries. Regular surveys in these countries are necessary, as the pest can appear at any time, and measures should be taken to minimize the potential negative impact of the future outbreak. Probabilistic processes, for example, weather phenomena, depend on many unpredictable factors, but it does not mean that their forecasting is useless. Forecasts for the near future are more reliable than for a long period. Several unpredictable factors listed below could in some circumstances slow or facilitate the spread of the pest. However, it is unlikely, that they will significantly change the rate of pest spread in the nearest 5 years. Unpredictable factors that could slow the spread of the pest are as follows:

- Extremely severe frosts in winter. The overwintering as prepupa is obligate for *A. planipennis* (Herms & McCullough, 2014; Orlova‐Bienkowskaja & Bieńkowski, 2016), and prepupae cannot survive temperature below −35.3°C (Crosthwaite, Sobek, Lyons, Bernards, & Sinclair, 2011). The minimum temperature in Moscow in the last 25 years was −30.8°C in 2006. But more severe frosts have sometimes happened in Moscow. For example, on 31 December 1978, the temperature in Moscow was −38°C (Meteoweb.ru, 2017). In the event of such frost, a population crash of *A. planipennis* in the northern part of its current range in European Russia is quite possible.
- The outbreak of parasitoids. About 50% of elder instars of *A. planipennis* are killed by the parasitoid *Spathius polonicus* (Orlova‐Bienkowskaja, 2015; Orlova‐Bienkowskaja & Belokobylskij, 2014). If the outbreak of this parasitoid occurs, this natural enemy can significantly suppress the population of *A. planipennis* and slow its spread.
- Spontaneous population crash. Substantial populations of invasive nonindigenous species occasionally collapse dramatically because of diseases and other causes (Simberloff & Gibbons, 2004).

Factors which could in theory facilitate the spread of the pest are as follows:

- Extremely hot summer. The extreme heat and drought could facilitate outbreaks of wood‐boring pests (Komarova, 2015). So if the summer will be extremely hot, it could facilitate the spread of *A*. *planipennis*.
- Spread to the forests. *Agrilus planipennis* has not yet spread from cities and roadsides to the forests of European Russia. In theory, the penetration to the forest could facilitate dispersal of the pest. But in reality, this possibility seems doubtful. As it has been mentioned above, native ash *F. excelsior* is not affected even in the forests situated close to the cities with severely damaged plantations of *F. pennsylvanica*.
- Ash dieback. This new severe disease of ash trees caused by the invasive ascomycete fungus *Hymenoscyphus fraxineus* originating from East Asia is spreading in Europe, and the ranges of *H*. *fraxineus* and *A. planipennis* have overlapped in European Russia (Musolin, Selikhovkin, Shabunin, Zviagintsev, & Baranchikov, 2017). There are still no studies on ecological interactions between these species. But since whole range of *A*. *planipennis* is in the region already occupied by *H*. *fraxineus* (Musolin et al., 2017), it seems unlikely that these interactions will significantly change the rate of spread of *A. planipennis*.

It is quite possible that the rate of disperse of *A. planipennis* in the south will be higher than in the north. The northernmost point of the native range is at the latitude 50°N (Orlova‐Bienkowskaja & Volkovitsh, 2017), and the northernmost point of current distribution (Yaroslavl) is situated in the latitude 58°N (Orlova‐Bienkowskaja, 2014b). So it is unknown, whether *A. planipennis* is able to disperse further north in Europe. Our model does not take this difference into account. It would be useful to compare our model based on just one parameter—distance between cities—with some models based on climatic factors and distribution of host plants. But we have no such possibility, as such models have not been elaborated. The spread of invasive organisms is a complex stochastic process depending of the heterogeneity of landscape. The date when the pest will reach a particular destination (for example, cross the western border of Russia) cannot be estimated intuitively, as it cannot be described by a “speed” value. In particular, *A. planipennis* spreads quickly in the region where cities are situated close to each other and slower in regions where cities are far from each other.

Dispersal of invasive pests is a stochastic process depending on many unknown factors. According to the theory of system dynamics by Forrester (1997), dynamics of such processes can be modeled in a general way only. The most realistic models of such systems are simple and do not include many factors into consideration. The more factors we consider, the higher the uncertainty and the worse the model. So only one factor—distance between the cities—was chosen for modeling. The elaborated model is simple, depict long‐distance dispersal in a very general way, do not deal with particular mechanisms of human‐mediated dispersal and do not take into account many factors, which could affect the dispersal of the pest: freight movement, general passenger traffic, population density, landscape, climate, etc. But we do not have enough information about the influence of each of these factors and about their interference. And the more factors that are included, the more assumptions are necessary, so the uncertainty is increased (Brockmann & Helbing, 2013). For example, the complex prognosis of spread of *A. planipennis* in North America taking into account the five factors (Iverson et al., 2010) was not realized (Siegert et al., 2015). Therefore, simple models are more appropriate. We believe that the simple approach—a recurrent algorithm with fat‐tailed kernel using just one parameter (pairwise distances between all locations)—could be applied for modeling not only *A. planipennis*, but also other invasive pests connected with urban landscapes.

## CONFLICT OF INTEREST

None declared.

## AUTHOR CONTRIBUTION

Authors work on the project “An integrative study of invasions in beetles.”

M.J.O.‐B. conceived the study and developed probabilistic models; both authors made field surveys and wrote the manuscript.

## DATA ACCESSIBILITY

All data supporting the results in the paper are included to Supporting Information available online with this article (Supporting information Appendices S1‐S5).
